# Crystal structure of azimsulfuron

**DOI:** 10.1107/S2056989015010968

**Published:** 2015-06-13

**Authors:** Youngeun Jeon, Jineun Kim, Eunjin Kwon, Tae Ho Kim

**Affiliations:** a Department of Chemistry and Research Institute of Natural Sciences, Gyeongsang National University, Jinju 660-701, Republic of Korea

**Keywords:** crystal structure, azimsulfuron, hydrogen bonding, herbicide

## Abstract

The title compound {systematic name: 1-(4,6-di­meth­oxy­pyrimidin-2-yl)-3-[1-methyl-4-(2-methyl-2*H*-tetra­zol-5-yl)pyrazol-5-ylsulfon­yl]urea}, C_13_H_16_N_10_O_5_S, is a sulfonyl­urea herbicide. In this compound, the dihedral angles between the planes of the central pyrazole and the terminal di­meth­oxy­pyrimidine and tetra­zole rings are 79.10 (8) and 17.21 (16)°, respectively. In the crystal, N—H⋯O hydrogen bonds link adjacent mol­ecules, forming *R*_2_^2^(8) inversion dimers. In addition, weak C—H⋯O and C—H⋯N hydrogen bonds and weak π–π inter­actions [ring centroid separation = 3.8255 (12) Å] are present, resulting in a three-dimensional architecture.

## Related literature   

For information on the herbicidal properties of the title compound, see: Valle *et al.* (2006 ▸); Boschin *et al.* (2007 ▸). For a related crystal structure, see: Chopra *et al.* (2004 ▸).
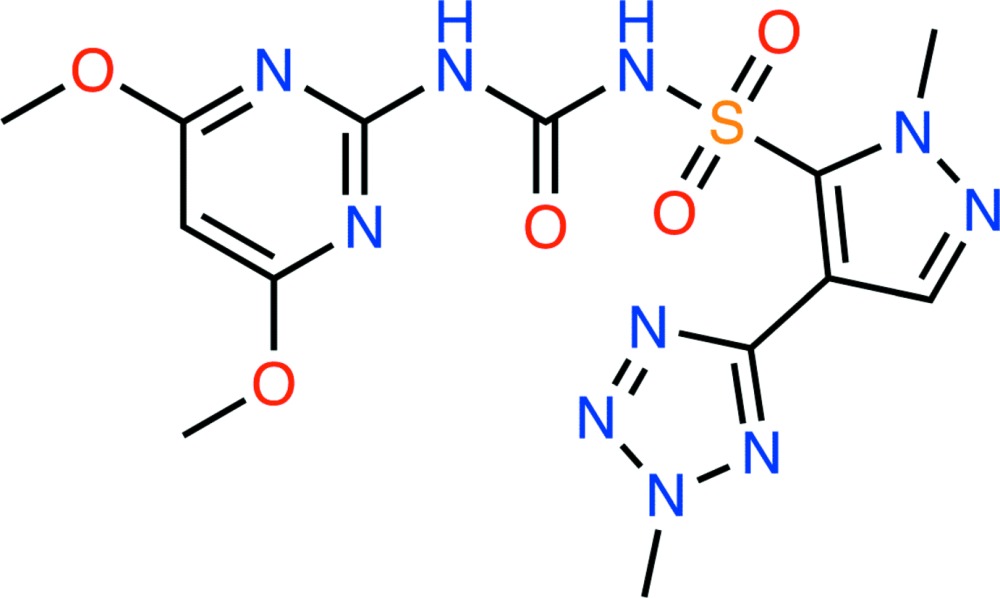


## Experimental   

### Crystal data  

C_13_H_16_N_10_O_5_S
*M* *_r_* = 424.42
Triclinic, 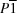

*a* = 8.5884 (7) Å
*b* = 9.9165 (7) Å
*c* = 12.2788 (13) Å
α = 73.190 (5)°
β = 75.819 (4)°
γ = 66.374 (3)°
*V* = 907.16 (14) Å^3^
*Z* = 2
Mo *K*α radiation
μ = 0.23 mm^−1^
*T* = 173 K
0.42 × 0.10 × 0.09 mm

### Data collection  

Bruker APEXII CCD diffractometer
Absorption correction: multi-scan (*SADABS*; Bruker, 2013 ▸) *T*_min_ = 0.909, *T*_max_ = 0.979
16122 measured reflections
4123 independent reflections
3357 reflections with *I* > 2σ(*I*)
*R*_int_ = 0.036

### Refinement  

*R*[*F* ^2^ > 2σ(*F* ^2^)] = 0.045
*wR*(*F* ^2^) = 0.118
*S* = 1.04
4123 reflections
266 parameters
H-atom parameters constrained
Δρ_max_ = 0.56 e Å^−3^
Δρ_min_ = −0.49 e Å^−3^

### 

Data collection: *APEX2* (Bruker, 2013 ▸); cell refinement: *SAINT* (Bruker, 2013 ▸); data reduction: *SAINT*; program(s) used to solve structure: *SHELXS97* (Sheldrick, 2008 ▸); program(s) used to refine structure: *SHELXL2013* (Sheldrick, 2015 ▸); molecular graphics: *SHELXTL* (Sheldrick, 2008 ▸); software used to prepare material for publication: *SHELXTL*.

## Supplementary Material

Crystal structure: contains datablock(s) global, I. DOI: 10.1107/S2056989015010968/hg5444sup1.cif

Structure factors: contains datablock(s) I. DOI: 10.1107/S2056989015010968/hg5444Isup2.hkl

Supporting information file. DOI: 10.1107/S2056989015010968/hg5444Isup3.cml

**Fig. 1.**
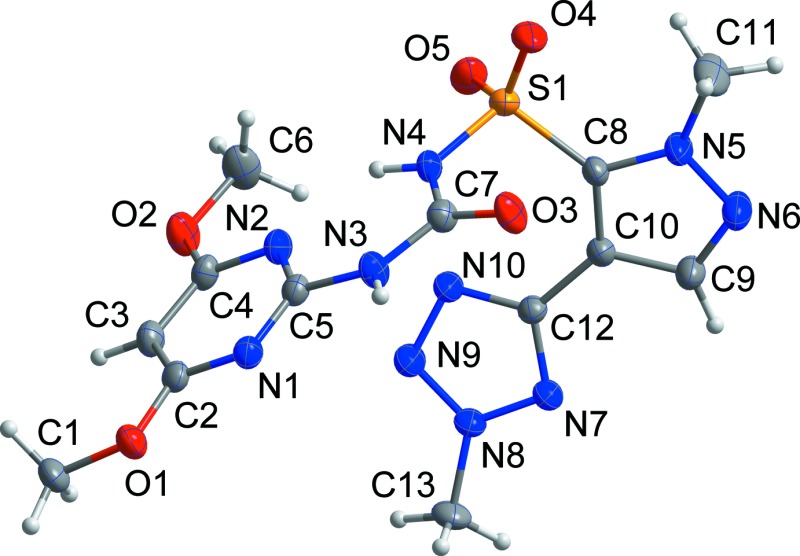
The asymmetric unit of the title compound with the atom numbering scheme. Displacement ellipsoids are drawn at the 50% probability level. H atoms are shown as small spheres of arbitrary radius.

**Fig. 2.**
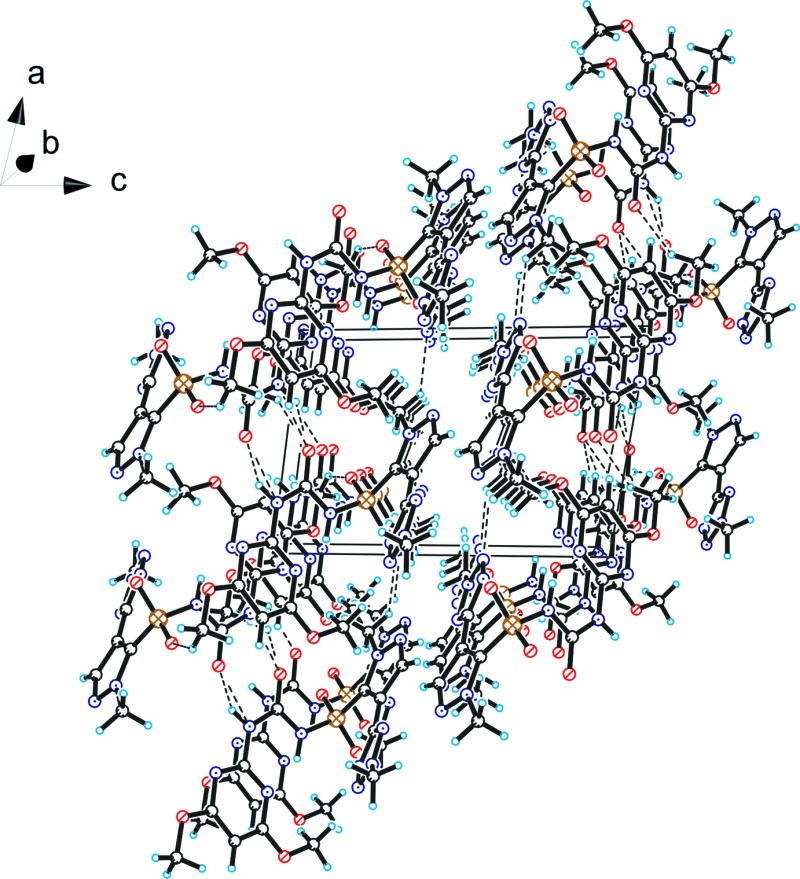
Crystal packing viewed along the b axis. The hydrogen bonds are shown as dashed lines.

. DOI: 10.1107/S2056989015010968/hg5444fig1.tif
The asymmetric unit of the title compound with the atom numbering scheme. Displacement ellipsoids are drawn at the 50% probability level. H atoms are shown as small spheres of arbitrary radius.

b . DOI: 10.1107/S2056989015010968/hg5444fig2.tif
Crystal packing viewed along the *b* axis. The hydrogen bonds are shown as dashed lines.

CCDC reference: 1405357

Additional supporting information:  crystallographic information; 3D view; checkCIF report

## Figures and Tables

**Table 1:** Hydrogen-bond geometry (, )

*D*H*A*	*D*H	H*A*	*D* *A*	*D*H*A*
N3H3*N*O3^i^	0.88	2.09	2.877(2)	149
C1H1*A*O3^ii^	0.98	2.55	3.381(3)	143
C1H1*C*O4^iii^	0.98	2.44	3.225(3)	137
C11H11*A*N9^iv^	0.98	2.57	3.357(3)	137
C13H13*B*N6^v^	0.98	2.62	3.533(3)	155

Symmetry codes: (i) 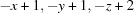; (ii) 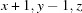; (iii) 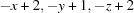; (iv) 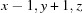; (v) 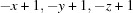.

## supplementary crystallographic information

### S1. Comment

Azimsulfuron [systematic name: 1-(4,6-dimethoxypyrimidin-2-yl)-3-[1-methyl-4-(2-methyl-2*H*-tetrazol-5-yl)pyrazol-5-ylsulfonyl]urea] is a sulfonylurea herbicide, a group of pesticides widely used all over the world for controlling weeds in several crops, rice, wheat, maize, barley, sugar beet, and tomato (Valle *et al.*, 2006). However, until now its crystal structure has not been reported. In the title compound (Fig. 1), the dihedral angles between the planes of the central pyrazol and the terminal dimethoxypyrimidinyl and tetrazol rings are 79.10 (8) and 17.21 (16)°, respectively. All bond lengths and bond angles are normal and comparable to those observed in similar crystal structures (Chopra *et al.*, 2004).

In the crystal structure (Fig. 2), molecules are linked by a pairs of N–H···O hydrogen bonds(Table 1), forming inversion dimmers with an *R*_2_^2^(8) ring motif. In addition, weak C–H···O and C–H···N hydrogen bonding and weak intermolecular π–π interactions between the terminal tetrazol ring systems [C*g*2···C*g*2^i^ = 3.8255 (12) Å] are present (C*g*2 is the centroid of the N7–N8–N9–N10–C12 ring) [for symmetry codes: (i), -*x*, -*y* + 1, -*z* + 1].

### S2. Experimental

The title compound was purchased from the Dr. Ehrenstorfer GmbH Company. Slow evaporation of a solution in CH_2_Cl_2_ gave single crystals suitable for X-ray analysis.

### S3. Refinement

All H-atoms were positioned geometrically and refined using a riding model with d(N—H) = 0.88 Å, *U*_iso_ = 1.2*U*_eq_(C) for urea N—H, d(C—H) = 0.98 Å, *U*_iso_ = 1.5*U*_eq_(C) for methyl group, d(C—H) = 0.95 Å, *U*_iso_ = 1.2*U*_eq_(C) for aromatic C—H.

### Crystal data

**Table:**

C_13_H_16_N_10_O_5_S	*Z* = 2
*M**_r_* = 424.42	*F*(000) = 440
Triclinic, *P*1	*D*_x_ = 1.554 Mg m^−^^3^
*a* = 8.5884 (7) Å	Mo *K*α radiation, λ = 0.71073 Å
*b* = 9.9165 (7) Å	Cell parameters from 4687 reflections
*c* = 12.2788 (13) Å	θ = 2.3–26.9°
α = 73.190 (5)°	µ = 0.23 mm^−^^1^
β = 75.819 (4)°	*T* = 173 K
γ = 66.374 (3)°	Block, colourless
*V* = 907.16 (14) Å^3^	0.42 × 0.10 × 0.09 mm

### Data collection

**Table:**

Bruker APEXII CCD diffractometer	3357 reflections with *I* > 2σ(*I*)
φ and ω scans	*R*_int_ = 0.036
Absorption correction: multi-scan (*SADABS*; Bruker, 2013)	θ_max_ = 27.5°, θ_min_ = 1.8°
*T*_min_ = 0.909, *T*_max_ = 0.979	*h* = −11→11
16122 measured reflections	*k* = −12→12
4123 independent reflections	*l* = −15→15

### Refinement

**Table:**

Refinement on *F*^2^	0 restraints
Least-squares matrix: full	Hydrogen site location: inferred from neighbouring sites
*R*[*F*^2^ > 2σ(*F*^2^)] = 0.045	H-atom parameters constrained
*wR*(*F*^2^) = 0.118	*w* = 1/[σ^2^(*F*_o_^2^) + (0.0543*P*)^2^ + 0.5222*P*] where *P* = (*F*_o_^2^ + 2*F*_c_^2^)/3
*S* = 1.04	(Δ/σ)_max_ < 0.001
4123 reflections	Δρ_max_ = 0.56 e Å^−^^3^
266 parameters	Δρ_min_ = −0.49 e Å^−^^3^

### Special details

**Table:**

Geometry. All e.s.d.'s (except the e.s.d. in the dihedral angle between two l.s. planes) are estimated using the full covariance matrix. The cell e.s.d.'s are taken into account individually in the estimation of e.s.d.'s in distances, angles and torsion angles; correlations between e.s.d.'s in cell parameters are only used when they are defined by crystal symmetry. An approximate (isotropic) treatment of cell e.s.d.'s is used for estimating e.s.d.'s involving l.s. planes.

### Fractional atomic coordinates and isotropic or equivalent isotropic displacement parameters (Å^2^)

**Table:**

	*x*	*y*	*z*	*U*_iso_*/*U*_eq_	
S1	0.74797 (6)	0.79478 (5)	0.73248 (4)	0.02370 (14)	
O1	1.10371 (18)	−0.02315 (15)	1.11503 (12)	0.0280 (3)	
O2	1.32890 (18)	0.30498 (17)	0.81805 (13)	0.0356 (4)	
O3	0.53148 (17)	0.65412 (16)	0.91451 (13)	0.0326 (4)	
O4	0.6527 (2)	0.91670 (16)	0.78851 (14)	0.0379 (4)	
O5	0.90400 (19)	0.79382 (17)	0.65785 (14)	0.0361 (4)	
N1	0.9372 (2)	0.21202 (18)	1.04786 (14)	0.0236 (4)	
N2	1.0436 (2)	0.38255 (18)	0.89699 (14)	0.0238 (4)	
N3	0.7525 (2)	0.44579 (18)	0.97760 (14)	0.0254 (4)	
H3N	0.6779	0.4182	1.0336	0.031*	
N4	0.8032 (2)	0.63736 (17)	0.82771 (13)	0.0214 (3)	
H4N	0.9133	0.5838	0.8284	0.026*	
N5	0.4566 (2)	0.88624 (18)	0.63630 (16)	0.0303 (4)	
N6	0.3789 (2)	0.8477 (2)	0.57414 (18)	0.0394 (5)	
N7	0.7377 (2)	0.40229 (17)	0.58472 (14)	0.0247 (4)	
N8	0.8855 (2)	0.28937 (17)	0.59920 (14)	0.0243 (4)	
N9	0.9977 (2)	0.32420 (18)	0.63093 (16)	0.0298 (4)	
N10	0.9232 (2)	0.46781 (18)	0.63767 (15)	0.0280 (4)	
C1	1.2673 (3)	−0.1457 (2)	1.11176 (19)	0.0308 (5)	
H1A	1.3074	−0.1629	1.0333	0.046*	
H1B	1.2544	−0.2373	1.1643	0.046*	
H1C	1.3512	−0.1201	1.1355	0.046*	
C2	1.0957 (2)	0.1075 (2)	1.04081 (16)	0.0228 (4)	
C3	1.2338 (2)	0.1330 (2)	0.96351 (17)	0.0262 (4)	
H3	1.3452	0.0575	0.9589	0.031*	
C4	1.1988 (2)	0.2757 (2)	0.89353 (17)	0.0244 (4)	
C5	0.9207 (2)	0.3420 (2)	0.97398 (16)	0.0215 (4)	
C6	1.2928 (3)	0.4514 (3)	0.7412 (2)	0.0393 (6)	
H6A	1.2067	0.4667	0.6948	0.059*	
H6B	1.3985	0.4573	0.6904	0.059*	
H6C	1.2486	0.5297	0.7861	0.059*	
C7	0.6855 (2)	0.5842 (2)	0.90723 (16)	0.0231 (4)	
C8	0.6097 (2)	0.7766 (2)	0.65845 (16)	0.0215 (4)	
C9	0.4833 (3)	0.7125 (2)	0.5569 (2)	0.0316 (5)	
H9	0.4601	0.6571	0.5153	0.038*	
C10	0.6314 (2)	0.6612 (2)	0.60716 (16)	0.0212 (4)	
C11	0.3620 (3)	1.0278 (3)	0.6757 (3)	0.0496 (7)	
H11A	0.2815	1.0973	0.6224	0.074*	
H11B	0.4431	1.0732	0.6783	0.074*	
H11C	0.2981	1.0074	0.7527	0.074*	
C12	0.7652 (2)	0.5127 (2)	0.60905 (16)	0.0204 (4)	
C13	0.9157 (3)	0.1370 (2)	0.5897 (2)	0.0326 (5)	
H13A	1.0396	0.0807	0.5778	0.049*	
H13B	0.8653	0.1424	0.5244	0.049*	
H13C	0.8625	0.0857	0.6606	0.049*	

### Atomic displacement parameters (Å^2^)

**Table:**

	*U*^11^	*U*^22^	*U*^33^	*U*^12^	*U*^13^	*U*^23^
S1	0.0261 (3)	0.0177 (2)	0.0299 (3)	−0.00848 (19)	−0.0094 (2)	−0.00349 (18)
O1	0.0263 (7)	0.0221 (7)	0.0311 (8)	−0.0045 (6)	−0.0073 (6)	−0.0024 (6)
O2	0.0173 (7)	0.0365 (8)	0.0414 (9)	−0.0065 (6)	−0.0006 (6)	0.0009 (7)
O3	0.0177 (7)	0.0287 (7)	0.0380 (8)	−0.0007 (6)	0.0005 (6)	−0.0025 (6)
O4	0.0478 (10)	0.0220 (7)	0.0494 (9)	−0.0038 (7)	−0.0234 (8)	−0.0146 (7)
O5	0.0301 (8)	0.0364 (8)	0.0425 (9)	−0.0205 (7)	−0.0075 (7)	0.0047 (7)
N1	0.0213 (8)	0.0249 (8)	0.0240 (8)	−0.0068 (7)	−0.0050 (7)	−0.0050 (6)
N2	0.0176 (8)	0.0255 (8)	0.0253 (8)	−0.0046 (7)	−0.0039 (7)	−0.0049 (7)
N3	0.0174 (8)	0.0254 (8)	0.0260 (8)	−0.0041 (7)	0.0002 (7)	−0.0024 (7)
N4	0.0162 (7)	0.0200 (7)	0.0251 (8)	−0.0028 (6)	−0.0050 (6)	−0.0042 (6)
N5	0.0265 (9)	0.0225 (8)	0.0423 (10)	0.0018 (7)	−0.0164 (8)	−0.0132 (7)
N6	0.0336 (10)	0.0331 (10)	0.0538 (12)	0.0015 (8)	−0.0243 (9)	−0.0174 (9)
N7	0.0234 (8)	0.0194 (7)	0.0312 (9)	−0.0041 (7)	−0.0068 (7)	−0.0078 (7)
N8	0.0260 (8)	0.0182 (7)	0.0293 (9)	−0.0047 (7)	−0.0063 (7)	−0.0087 (6)
N9	0.0252 (9)	0.0236 (8)	0.0411 (10)	−0.0021 (7)	−0.0102 (8)	−0.0131 (7)
N10	0.0233 (8)	0.0212 (8)	0.0409 (10)	−0.0024 (7)	−0.0103 (8)	−0.0121 (7)
C1	0.0283 (11)	0.0193 (9)	0.0403 (12)	−0.0001 (8)	−0.0127 (9)	−0.0055 (8)
C2	0.0234 (10)	0.0227 (9)	0.0236 (9)	−0.0058 (8)	−0.0094 (8)	−0.0056 (7)
C3	0.0180 (9)	0.0271 (10)	0.0298 (10)	−0.0027 (8)	−0.0066 (8)	−0.0060 (8)
C4	0.0165 (9)	0.0297 (10)	0.0262 (10)	−0.0071 (8)	−0.0042 (8)	−0.0056 (8)
C5	0.0173 (9)	0.0246 (9)	0.0228 (9)	−0.0049 (8)	−0.0044 (7)	−0.0076 (7)
C6	0.0283 (11)	0.0411 (13)	0.0386 (13)	−0.0145 (10)	0.0007 (10)	0.0043 (10)
C7	0.0200 (9)	0.0242 (9)	0.0230 (9)	−0.0051 (8)	−0.0010 (8)	−0.0075 (7)
C8	0.0213 (9)	0.0172 (8)	0.0249 (9)	−0.0028 (7)	−0.0079 (8)	−0.0050 (7)
C9	0.0297 (11)	0.0264 (10)	0.0419 (12)	−0.0028 (9)	−0.0170 (10)	−0.0124 (9)
C10	0.0209 (9)	0.0187 (9)	0.0237 (9)	−0.0057 (7)	−0.0056 (8)	−0.0040 (7)
C11	0.0405 (14)	0.0317 (12)	0.0737 (18)	0.0128 (11)	−0.0255 (13)	−0.0302 (12)
C12	0.0206 (9)	0.0198 (8)	0.0219 (9)	−0.0056 (7)	−0.0053 (7)	−0.0066 (7)
C13	0.0393 (12)	0.0181 (9)	0.0415 (12)	−0.0063 (9)	−0.0074 (10)	−0.0125 (8)

### Geometric parameters (Å, º)

**Table:**

S1—O4	1.4201 (15)	N8—N9	1.314 (2)
S1—O5	1.4243 (16)	N8—C13	1.462 (2)
S1—N4	1.6284 (16)	N9—N10	1.325 (2)
S1—C8	1.751 (2)	N10—C12	1.350 (2)
O1—C2	1.338 (2)	C1—H1A	0.9800
O1—C1	1.444 (2)	C1—H1B	0.9800
O2—C4	1.337 (2)	C1—H1C	0.9800
O2—C6	1.444 (3)	C2—C3	1.387 (3)
O3—C7	1.217 (2)	C3—C4	1.383 (3)
N1—C5	1.321 (2)	C3—H3	0.9500
N1—C2	1.341 (2)	C6—H6A	0.9800
N2—C4	1.330 (2)	C6—H6B	0.9800
N2—C5	1.342 (2)	C6—H6C	0.9800
N3—C7	1.373 (2)	C8—C10	1.389 (3)
N3—C5	1.397 (2)	C9—C10	1.393 (3)
N3—H3N	0.8800	C9—H9	0.9500
N4—C7	1.378 (2)	C10—C12	1.459 (2)
N4—H4N	0.8800	C11—H11A	0.9800
N5—N6	1.340 (3)	C11—H11B	0.9800
N5—C8	1.358 (2)	C11—H11C	0.9800
N5—C11	1.466 (3)	C13—H13A	0.9800
N6—C9	1.325 (3)	C13—H13B	0.9800
N7—N8	1.324 (2)	C13—H13C	0.9800
N7—C12	1.332 (2)		
			
O4—S1—O5	119.58 (10)	N2—C4—C3	123.37 (18)
O4—S1—N4	109.86 (9)	O2—C4—C3	117.43 (17)
O5—S1—N4	104.30 (9)	N1—C5—N2	127.89 (17)
O4—S1—C8	107.79 (9)	N1—C5—N3	114.04 (17)
O5—S1—C8	109.46 (10)	N2—C5—N3	118.05 (16)
N4—S1—C8	104.90 (9)	O2—C6—H6A	109.5
C2—O1—C1	117.09 (16)	O2—C6—H6B	109.5
C4—O2—C6	118.04 (16)	H6A—C6—H6B	109.5
C5—N1—C2	114.99 (17)	O2—C6—H6C	109.5
C4—N2—C5	114.96 (16)	H6A—C6—H6C	109.5
C7—N3—C5	129.98 (17)	H6B—C6—H6C	109.5
C7—N3—H3N	115.0	O3—C7—N3	121.66 (18)
C5—N3—H3N	115.0	O3—C7—N4	122.49 (17)
C7—N4—S1	123.00 (13)	N3—C7—N4	115.85 (16)
C7—N4—H4N	118.5	N5—C8—C10	107.00 (17)
S1—N4—H4N	118.5	N5—C8—S1	122.90 (14)
N6—N5—C8	111.29 (16)	C10—C8—S1	130.02 (14)
N6—N5—C11	117.40 (17)	N6—C9—C10	111.85 (19)
C8—N5—C11	131.12 (18)	N6—C9—H9	124.1
C9—N6—N5	105.78 (17)	C10—C9—H9	124.1
N8—N7—C12	101.76 (15)	C8—C10—C9	104.07 (16)
N9—N8—N7	113.84 (15)	C8—C10—C12	131.15 (18)
N9—N8—C13	123.11 (16)	C9—C10—C12	124.59 (18)
N7—N8—C13	122.87 (17)	N5—C11—H11A	109.5
N8—N9—N10	106.55 (15)	N5—C11—H11B	109.5
N9—N10—C12	105.48 (16)	H11A—C11—H11B	109.5
O1—C1—H1A	109.5	N5—C11—H11C	109.5
O1—C1—H1B	109.5	H11A—C11—H11C	109.5
H1A—C1—H1B	109.5	H11B—C11—H11C	109.5
O1—C1—H1C	109.5	N7—C12—N10	112.36 (16)
H1A—C1—H1C	109.5	N7—C12—C10	121.13 (17)
H1B—C1—H1C	109.5	N10—C12—C10	126.48 (17)
O1—C2—N1	112.43 (17)	N8—C13—H13A	109.5
O1—C2—C3	124.43 (17)	N8—C13—H13B	109.5
N1—C2—C3	123.14 (18)	H13A—C13—H13B	109.5
C4—C3—C2	115.60 (17)	N8—C13—H13C	109.5
C4—C3—H3	122.2	H13A—C13—H13C	109.5
C2—C3—H3	122.2	H13B—C13—H13C	109.5
N2—C4—O2	119.19 (17)		
			
O4—S1—N4—C7	59.80 (18)	C5—N3—C7—N4	−7.5 (3)
O5—S1—N4—C7	−170.87 (15)	S1—N4—C7—O3	−2.7 (3)
C8—S1—N4—C7	−55.81 (17)	S1—N4—C7—N3	176.66 (13)
C8—N5—N6—C9	0.1 (3)	N6—N5—C8—C10	0.1 (2)
C11—N5—N6—C9	−175.4 (2)	C11—N5—C8—C10	174.9 (2)
C12—N7—N8—N9	0.4 (2)	N6—N5—C8—S1	177.18 (16)
C12—N7—N8—C13	175.78 (18)	C11—N5—C8—S1	−8.1 (3)
N7—N8—N9—N10	−0.4 (2)	O4—S1—C8—N5	15.8 (2)
C13—N8—N9—N10	−175.73 (18)	O5—S1—C8—N5	−115.78 (18)
N8—N9—N10—C12	0.2 (2)	N4—S1—C8—N5	132.80 (17)
C1—O1—C2—N1	177.72 (17)	O4—S1—C8—C10	−167.94 (18)
C1—O1—C2—C3	−1.3 (3)	O5—S1—C8—C10	60.5 (2)
C5—N1—C2—O1	−178.07 (16)	N4—S1—C8—C10	−50.9 (2)
C5—N1—C2—C3	1.0 (3)	N5—N6—C9—C10	−0.4 (3)
O1—C2—C3—C4	179.64 (18)	N5—C8—C10—C9	−0.3 (2)
N1—C2—C3—C4	0.7 (3)	S1—C8—C10—C9	−177.09 (17)
C5—N2—C4—O2	178.90 (17)	N5—C8—C10—C12	−175.5 (2)
C5—N2—C4—C3	−0.2 (3)	S1—C8—C10—C12	7.8 (3)
C6—O2—C4—N2	−1.7 (3)	N6—C9—C10—C8	0.4 (3)
C6—O2—C4—C3	177.50 (19)	N6—C9—C10—C12	176.0 (2)
C2—C3—C4—N2	−1.1 (3)	N8—N7—C12—N10	−0.3 (2)
C2—C3—C4—O2	179.76 (18)	N8—N7—C12—C10	−178.32 (17)
C2—N1—C5—N2	−2.7 (3)	N9—N10—C12—N7	0.1 (2)
C2—N1—C5—N3	175.99 (17)	N9—N10—C12—C10	177.97 (18)
C4—N2—C5—N1	2.3 (3)	C8—C10—C12—N7	159.2 (2)
C4—N2—C5—N3	−176.29 (17)	C9—C10—C12—N7	−15.0 (3)
C7—N3—C5—N1	−173.08 (19)	C8—C10—C12—N10	−18.5 (3)
C7—N3—C5—N2	5.7 (3)	C9—C10—C12—N10	167.3 (2)
C5—N3—C7—O3	171.9 (2)		

### Hydrogen-bond geometry (Å, º)

**Table:**

*D*—H···*A*	*D*—H	H···*A*	*D*···*A*	*D*—H···*A*
N3—H3*N*···O3^i^	0.88	2.09	2.877 (2)	149
C1—H1*A*···O3^ii^	0.98	2.55	3.381 (3)	143
C1—H1*C*···O4^iii^	0.98	2.44	3.225 (3)	137
C11—H11*A*···N9^iv^	0.98	2.57	3.357 (3)	137
C13—H13*B*···N6^v^	0.98	2.62	3.533 (3)	155

Symmetry codes: (i) −*x*+1, −*y*+1, −*z*+2; (ii) *x*+1, *y*−1, *z*; (iii) −*x*+2, −*y*+1, −*z*+2; (iv) *x*−1, *y*+1, *z*; (v) −*x*+1, −*y*+1, −*z*+1.
