# Supplementary figures and images for: Crystal structure of azimsulfuron
Source: Acta Crystallogr E Crystallogr Commun. 2015 Jun 13;71(Pt 7):o470–1. doi: 10.1107/S2056989015010968 (PMC4518981; doi:10.1107/S2056989015010968)

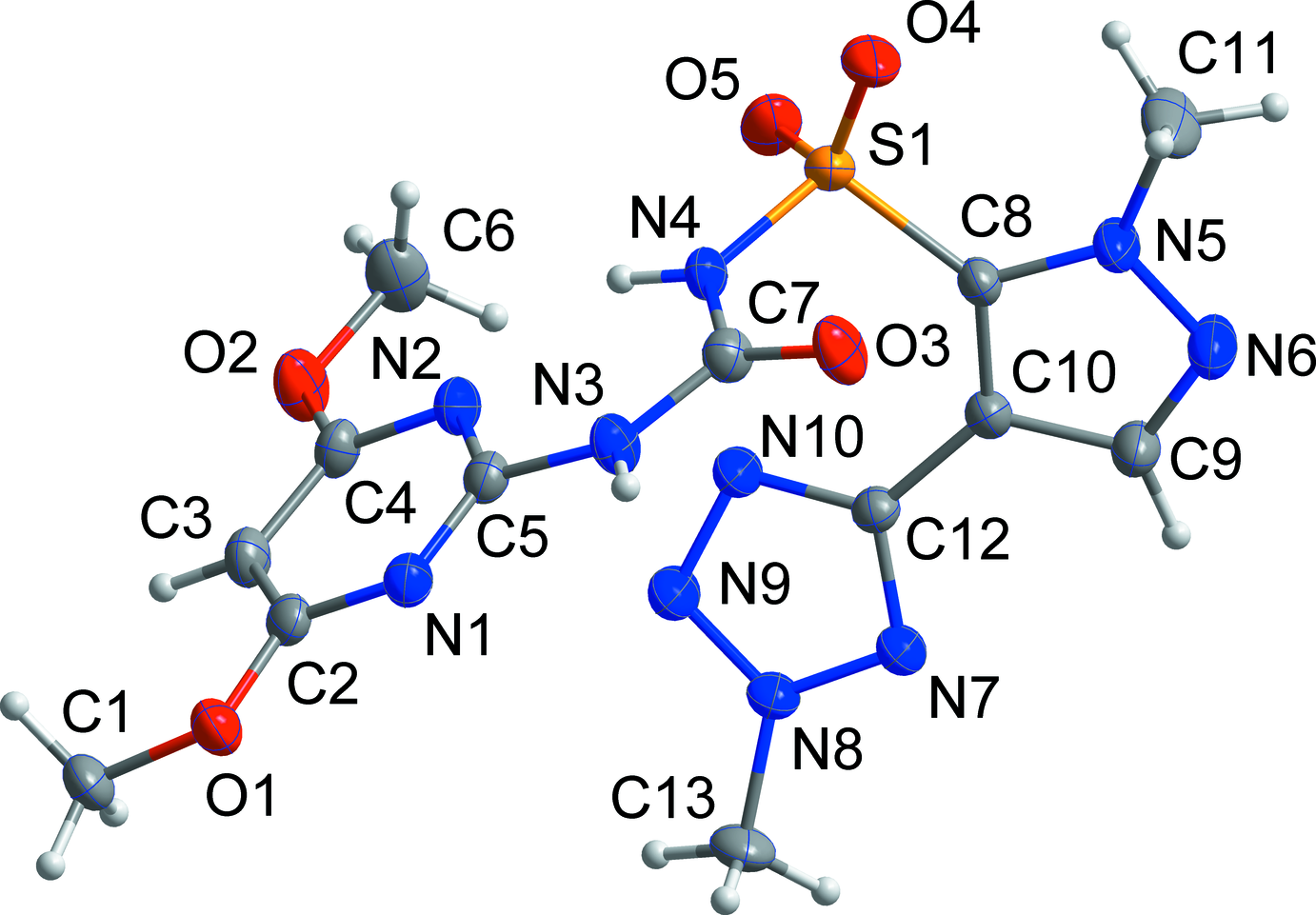

Supplement: Supplementary file 4 [file e-71-0o470-fig1.tif]

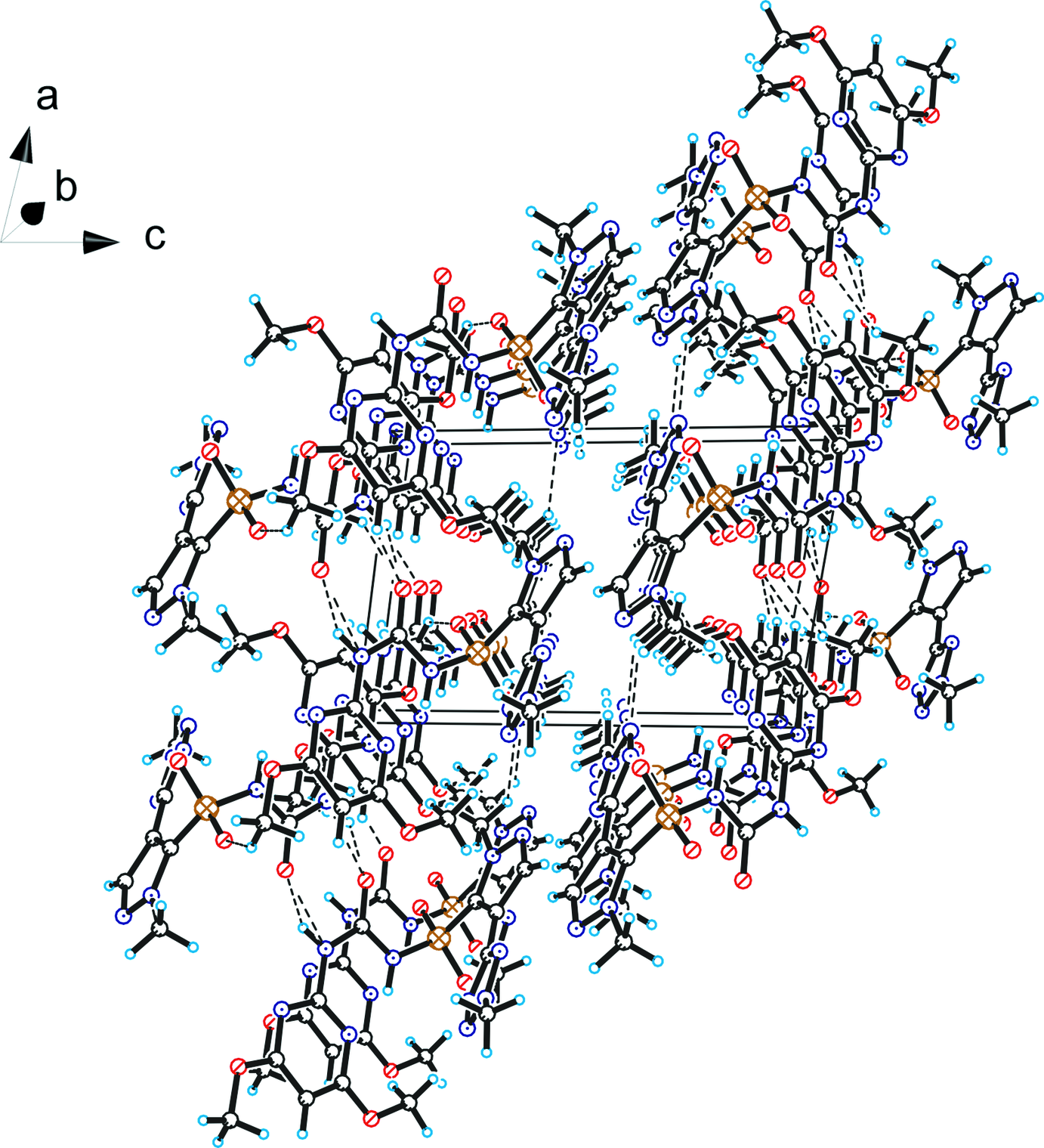

Supplement: Supplementary file 5 [file e-71-0o470-fig2.tif]
